# Effect of GnRH immunocastration on immune function in male rats

**DOI:** 10.3389/fimmu.2022.1023104

**Published:** 2023-01-13

**Authors:** Fuqiang Pan, Huiting Du, Weiguo Tian, Huihui Xie, Bochao Zhang, Wanzhen Fu, Yunsheng Li, Yinghui Ling, Yunhai Zhang, Fugui Fang, Ya Liu

**Affiliations:** ^1^ Department of Veterinary Medicine, College of Animal Science and Technology, Anhui Agricultural University, Hefei, Anhui, China; ^2^ Anhui Provincial Key Laboratory of Local Livestock and Poultry Genetical Resource Conservation and Breeding, College of Animal Science and Technology, Anhui Agricultural University, Hefei, Anhui, China; ^3^ Linquan County Modern Agriculture Technology Cooperation and Extension Service Center, Fuyang, Anhui, China

**Keywords:** GnRH, immunocastration, thymus, spleen, immune function, male rats

## Abstract

The present study aimed to reveal the effects of immunocastration on the development of the immune system in rats. Seventy rats were randomly assigned into two groups: Control (n = 35) and immunized (n = 35). Twenty-day-old rats were immunized with gonadotropin-releasing hormone (GnRH) and booster immunization was administered every two weeks (three immunizations in total). From 20-day-old rats, we collected samples every two weeks, including five immunized rats and five control rats (seven collections in total). We collected blood samples, testicles, thymuses, and spleens. The results showed that GnRH immunization increased the GnRH antibody titers and reduced the testosterone concentration (both *P < 0.05*). Compared with the control group, the number of CD4+CD8− cells, CD4−CD8+ cells, and CD4+CD8+ cells increased (*P < 0.05*) whereas the number of CD4-CD8- cells and CD4+CD25+ cells reduced in the immunized group (*P < 0.05*) over time. GnRH immunization also increased the relative weights of thymus and spleen (*P < 0.05*), serum concentrations of interleukin (IL)-2, IL-4, IL-6, IL-10, IL-17 and Interferon-γ (IFN-γ) over time (*P < 0.05*), and changed the mRNA levels of *IL-2, IL-4, IL-6. IL-10, IL-17, IFN-γ, CD4, D8, CD19 GnRH, and GnRH receptor* (*GnRH-R*) in thymus and spleen. Thus, GnRH immunization enhanced the immune markers in thymus, spleen, and blood immune cytokines in rats.

## 1 Introduction

It is known there is a close relationship between the hypothalamic-pituitary-gonadal axis (HPGA) and the immune system, and they regulate the development and functions reciprocally through the hormones and cytokines (1–6). Gonadotropin-releasing hormone (GnRH) is a pivotal hormone of HPGA, and it regulates reproduction through the HPGA. In the past decades, GnRH has been used as an immunocastration antigen in pigs (7–11), calf (12–14), goats (15–17), chickens (18), horses (19–21), and some wild animals (22, 23) to reduce their aggression, increasing yield, and improve the meat quality (24) or to control the reproduction of wild animals (22, 23). Many studies have confirmed that GnRH has impacts on the development and functions of immune system (3, 25). Blocking LHRH receptors during the critical period of brain-thymus-lymphoid axis dramatically impairs the development of the immune system (26). Activating GnRH receptor (GnRHR) impairs the ability of Tregs to inhibit conventional T cell proliferation (27). GnRH agonist transiently induce down-regulation of CD3 +T cell and CD25 + T cells subpopulation in peripheral blood (28) and stimulate the differentiation of CD69 + T cells (29). In thymus, GnRH regulates the proliferation of various subsets of T lymphocytes, the activation of natural killer cells and the synthesis of cytokines (30–33). GnRH agonist prevents the apoptosis of lymphocytes and attenuates the lipopolysaccharide (LPS) induced thymic atrophy in mice (34).GnRH also increases the percentages of CD4+ T lymphocytes in immunocompromised rats, and may participate in regulating the proliferation, activation, or functions of T helper cells (35).

Since immunization against GnRH can neutralize the GnRH in the body, and GnRH regulates immune system, so we speculate that GnRH immunization may affect the development and function of immune system in immunized animals. In fact, recent researches have verified our hypothesis. When immunized against GnRH, thymulin was downregulated in early stage and subsequently upregulated in male rats (36). Accordingly, T lymphocytes differentiation in the thymus was repressed in early stage, and then restored and enhanced at later time points (37). Immunization against GnRH also improves the splenic immune markers and serum cytokines (15), induces stronger lymphocyte proliferative responses and higher levels of IFN-γ (38).GnRH Immunocastration is gradually replacing surgical castration (39), however, little is known about the effects of GnRH active immunization on the immune system in immunized animals. The aim of the present study was to evaluate the effects of GnRH active immunization on the development of rats’ thymus and spleen, composition of T-cell subpopulation in peripheral blood, and serum level of some cytokines, and these results will help us to know how GnRH immunization affects the immune system in male mammalians.

## 2 Materials and methods

### 2.1 Vaccine and animals

The sequence of GnRH2 was designed according to Oonk’s description (40). One group of rats (immunization) received GnRH2 mixed with 50% adjuvant (MONTANIDE™ ISA201 VG, SEPPIC) saline solution, with 100 µg (0.5 mL) each; and the other group (control) received 50% adjuvant saline solution in same volume (37).

Seventy healthy male Sprague Dawley (SD) rats, average body weight 40 ± 6 g, were randomly divided into the immunized group (n = 35) and the control group (n = 35), and placed in a greenhouse (23 ~ 26 °C), with a 12 h light and 12 h dark cycle, commercial rat food and water were available ad libitum. All procedures involving animals were approved by the Animal Care and Use Committee of Anhui Agricultural University.

### 2.2 Experimental design and immunization procedure

At 20 days old, 35 rats in the immunized group were injected with the first vaccine, and the remaining five rats were sampled for 20 days. Afterwards, the rats received booster immunizations once every two weeks, twice. Rats were injected subcutaneously at four sites on their backs (37). Starting with the rats at 20 days old, five rats were sampled every two weeks in each group. At the last sampling point the rats were 104 days old, which was 12 weeks after the first immunization.

### 2.3 Collection of samples

Rats were weighed and their blood was collected before sacrifice (all rats from the last sample were weighed at each sampling point). A first blood sample (1 mL) was collected into a tube containing an anticoagulant for flow cytometry analysis; a second sample was collected into a tube containing no anticoagulant tube for enzyme-linked immunosorbent assays (ELISAs), both samples stored at 4 °C. The sacrificed rats were dissected and their testicles, thymus, and spleen were removed and weighed. We cut three piece of thymus and spleen respectively and placed them in cryoprotectant tubes then stored at −80 °C. The remaining part of the thymus and spleen stored at 4% paraformaldehyde, with the corresponding mark, together with the testicles.

### 2.4 Detection of GnRH antibody, testosterone and cytokines

Anti-GnRH antibodies (IgG) (DRE60558Ra, Shanghai Lianshuo Biological Technology Co., LTD) and the concentrations of testosterone (CK-E30426R) interleukin 2 (IL-2)(CK-E30648R), IL-4 (CK-E30647R), IL-6(CK-E30646R), IL-10(CK-E30651R), IL-17(CK-E30480R), tumor necrosis factor-alpha (TNF-α)(CK-E30635R), and interferon gamma (IFN-γ) (CK-E30654R) were measured using ELISA Kits (Shanghai Aoji Biotech Co., Ltd.). All experiments were conducted by following manufacturer’s instructions.

### 2.5 Blood immune cell profiles

The antibodies used here were as follows: Anti-Rat CD45 (percp-cy5.5) (OX-1 clone) (202220, BioLegend), Anti-Rat CD3 (PE) (eBioG4.18 clone) (12-0030-81, eBioscience), Anti-Rat CD4 (FITC) (OX35 clone) (11-0040-81, eBioscience), Anti-Rat CD8a (APC) (OX8 clone) (17-0084-82, eBioscience), Anti-Rat CD25 (APC) (OX39 clone) (17-0390-82, eBioscience), Anti-CD19 antibody (EPR2230(N) clone) (ab197895, abcam), Goat Anti-Rabbit IgG H&L (FITC) (Polyclonal clone) (ab6717, abcam), Mouse IgG1 K–APC (P3.6.2.8.1 clone) (17-4714-41, eBioscience), Mouse IgG1 K–FITC (P3.6.2.8.1 clone) (11-4714-41, eBioscience).

Whole blood was tested within 8 hours on the flow machine (FACSCalibur, America BD). Each rat blood sample was divided into three tubes and analyzed in parallel: A, CD45+CD3+CD4+CD8+; B, CD45+CD3+CD4+CD25+, C: CD45+CD3+CD19+. Anticoagulated blood (100 μL) was added to each tube and then 10 times the volume of red blood cell lysis buffer (BioGems) was added. The tubes were shaken and left to stand for 10 min. The tubes were then centrifuged for 5 min at 500 ×g and the supernatant was discarded. The cells were resuspended in 200 μL of phosphate-buffered saline (PBS). The corresponding antibody was added and the cells were incubated for 30 min. Thereafter, 500 μL of PBS was added and the cells were subjected to flow cytometry (FACSCalibur). Two additional blood samples were taken and two isotype controls (Mouse IgG1 K-FITC, Mouse IgG1 K-APC) were used to replace CD4, CD19, and CD8. Using CD45 for the gate, 10,000 cells are measured per blood sample.

### 2.6 Morphological observation of tissue samples

Tissues collected from the rats were fixed in 4% paraformaldehyde for 12 ~ 24 h at 4 °C, then were processed and stained with hematoxylin-eosin (HE) as Jiang et al. described (41) The morphological changes and tissue structure of testis, thymus and spleen were observed under light microscope (BX51). Images were taken with Nikon Digital Sight DS-SMC camera.

### 2.7 Analysis of mRNA expression

Tissue and cells RNA was extracted using Total RNA Kit II kit (R6934-01, R6831-01, OMEGA). Reverse transcription was performed according to the instructions of the reverse-transcription kit (R223-01, Nanjing Nuoweizan Biotech Co., Ltd.). The resulting cDNA samples were stored at -20 °C. Based on the cDNA sequences of *CD4*, *CD8*, *CD19*, *CD25*, *IL2*, *IL4*, *IL6*, *IL10*, *IL17*, *TNF-α*, *IFN-γ*, *GnRH*, *GnRH-R*, *AR*, and the reference rat β-actin gene in the GenBank database, primers (**Supplementary Table 1**) were designed using Primer Premier 5.0 and AlleleID 6.0 Synthesis by Shanghai Sangon Biotech Co., Ltd. Quantitative Real-time PCR was then performed using SYBR Green I as a fluorescent dye and real-time monitoring of fluorescence signal intensity. The reaction conditions were 40 cycles of 94 °C for 30 s, 94 °C for 5 s, and 60 °C for 30 s.

### 2.8 Statistical analyses

Validation data for the quantitative results were calculated, using the 2^-ΔΔCt^ method for statistical analysis. The relative expression of the target gene is F = 2^-ΔΔCT^, where ΔΔCt = The change in cycle threshold (Ct) value of the target gene − the ΔCt value of the reference gene. The results, as a value of F, were calculated by relative quantification for the significance analysis. The difference of anti-GnRH antibody titer, testosterone and cytokines between the immunized group and the control group was verified by paired sample t-test. (odds ratio (OR) and their 95% confidence intervals (CI) are reported). An alpha level of 0.05 was used for all statistical tests. All data were analyzed statistically using JMP 9.0. The results are shown as the mean ± the standard error of the mean (SEM). P<0.05 indicates a significant difference. The results were plotted as histograms using Graphpad 5.0.

## 3 Results

### 3.1 GnRH antibody titer

**Figure 1:**
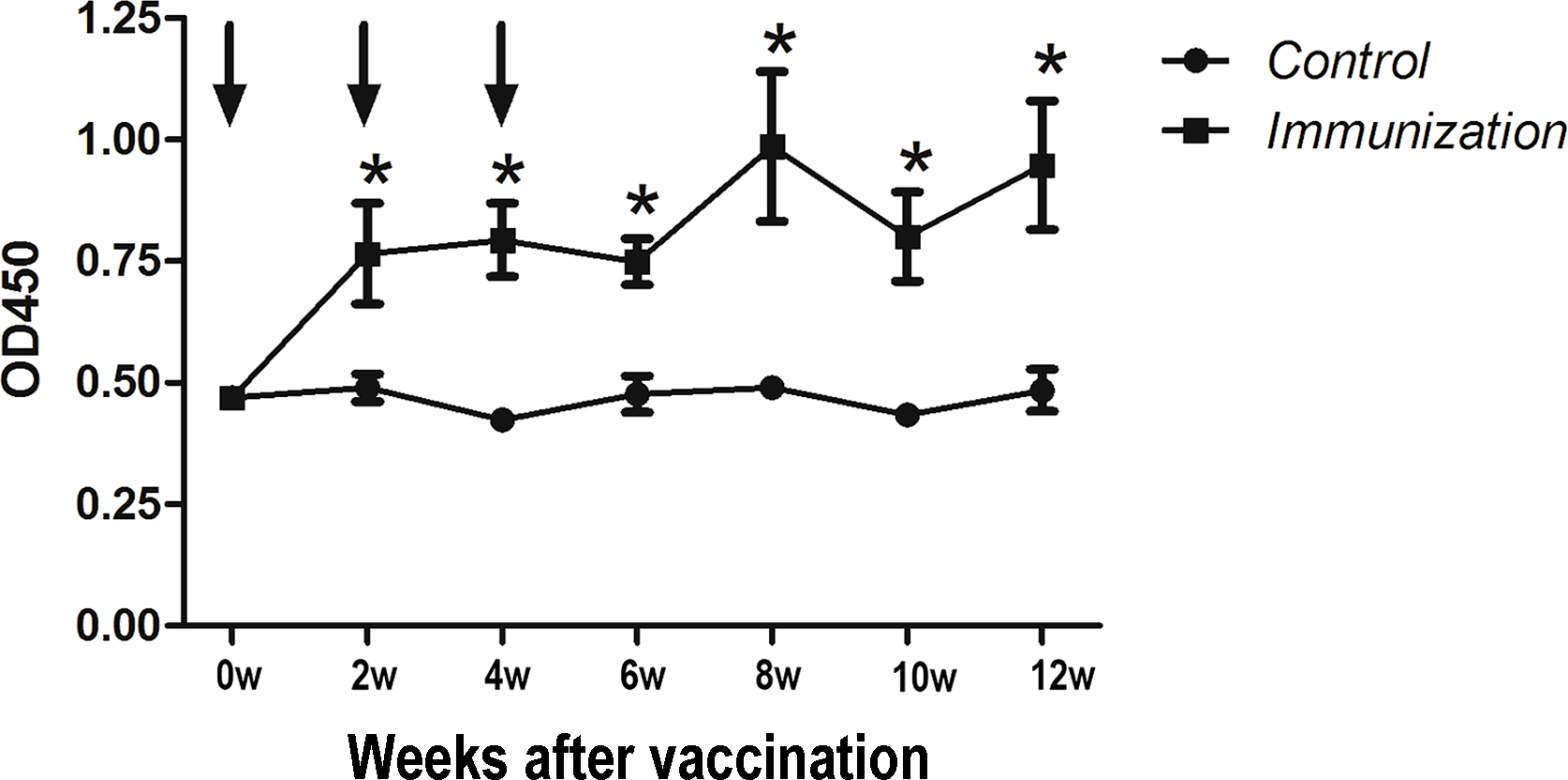
Mean (± SEM) of the serum antibody titers in the gonadotropin-releasing hormone (GnRH) immunization group (n = 35) and control group (n = 35). The ordinate is the OD of the serum GnRH antibody. The abscissa is the age of weeks after first immunization and Arrows indicate the primary vaccination and subsequent boosters. * Indicates a significant difference (*P* < 0.05).

Starting from the first immunization, anti-GnRH antibodies(IgG) were detected every two weeks (seven times in total) (**Figure 1**). Arrows indicate the time of the three immunizations. The results showed that after the first immunization, the antibody titer of the immunized group was significantly higher than that of the control group (*P < 0.05*) until the end of the study.

### 3.2 Impacts on testicular development and serum testosterone

**Figure 2:**
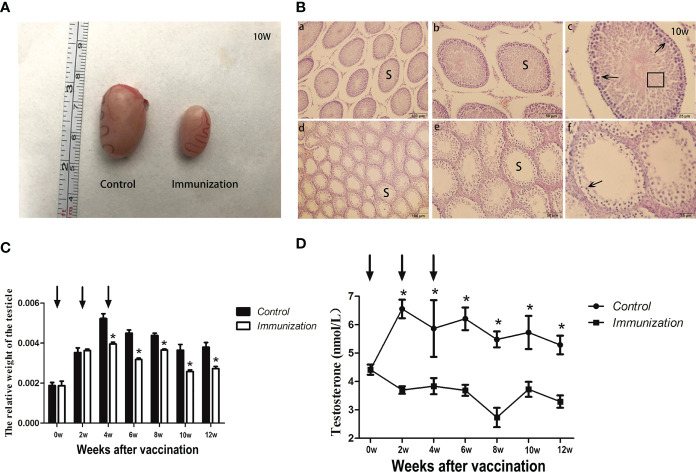
**(A)** Images of testicles of the gonadotropin-releasing hormone (GnRH) immunization and control groups from 10 Week rats after first immunization. **(B)** Histopathological observation of testes samples from the control group (a, b, c), and the gonadotropin-releasing hormone (GnRH) immunization group (d, e, f) at 10 weeks after first immunizaton. (a-c) and (d-f) represents different magnifications (100, 200, and 400 times, respectively). S indicates seminiferous tubules; Sperm cells at various stages (arrows), and spermatozoa (square area) are indicated. **(C)** Mean ( ± SEM) the relative weight of the testicle in gonadotropin-releasing hormone (GnRH) immunization group (n=35) and control group (n=35). Arrows indicate the primary vaccination and subsequent boosters. * Indicates a significant difference (*P* < 0.05). **(D)** Mean (± SEM) of the serum testosterone concentration in the gonadotropin-releasing hormone (GnRH) immunization group (n =35) and control group (n = 35). The ordinate is the concentration of testosterone. The abscissa is the age of weeks after first immunization and arrows indicate the primary vaccination and subsequent boosters. The testosterone levels were detected using an enzyme-linked immunosorbent assay (ELISA). * Indicates a significant difference (*P* < 0.05).

The testicles sampled when the rats were 90 days old showed that those of the immunized rats were significantly smaller than those of the control group (**Figure 2A**). The testicle seminiferous tubules of the rats in control group developed normally, intraluminal spermatogonia developed in the lumen and differentiated into obvious spermatogonia, primary spermatocytes, secondary spermatocytes, sperm cells, and sperm, with the cells arranged closely. In the immunized group, the cells inside the lumen were sparse, there were few cells of each stage of development, and almost no sperm (**Figure 2B**). At each sampling point, we calculated the relative weight of the testicle. At two weeks after the second immunization, the relative weight of the testicle of the immunized group was significantly lower than that of the control group (*P<0.05*) until the end of the study (**Figure 2C**). After the first immunization, the concentration of serum testosterone in the immunized group was significantly lower than that in the control group (*P < 0.05*) until the end of the study (**Figure 2D**). This indicted that GnRH immunization exerted its immunocastration function.

### 3.3 Serum cytokine profiles

**Figure 3:**
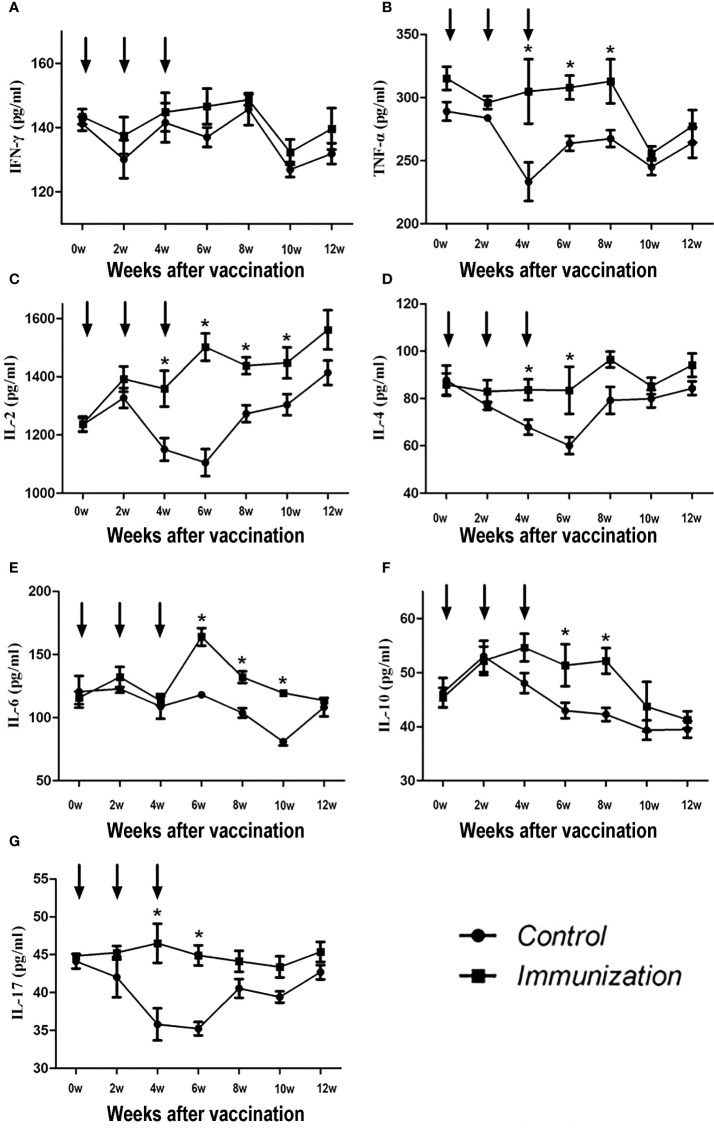
Mean (± SEM) of the serum IFN-γ **(A)**, TNF-α **(B)**, IL-2 **(C)**, IL-4 **(D)**, IL-6 **(E)**, IL-10 **(F)**, and IL-17 **(G)** concentrations in the gonadotropin-releasing hormone (GnRH) immunization group (n = 35) and control group (n = 35). All were detected using enzyme-linked immunosorbent assays (ELISAs). The ordinate is the concentration of immue cytokine. The abscissa is the age of weeks after first immunization and arrows indicate the primary vaccination and subsequent boosters. * Indicates a significant difference (*P*<0.05).

The concentration of IFN-γ showed no difference over the whole experimental period (*P >*0.05, **Figure 3A**). For the other factors, after the third immunization their levels were significantly higher in the immunized group compared with that in the control group (*P < 0.05*). However, over time, the serum cytokine levels in the immunized group and the control group tended to be similar (**Figures 3B-G**).

### 3.4 Blood immune cells profiles

**Figure 4:**
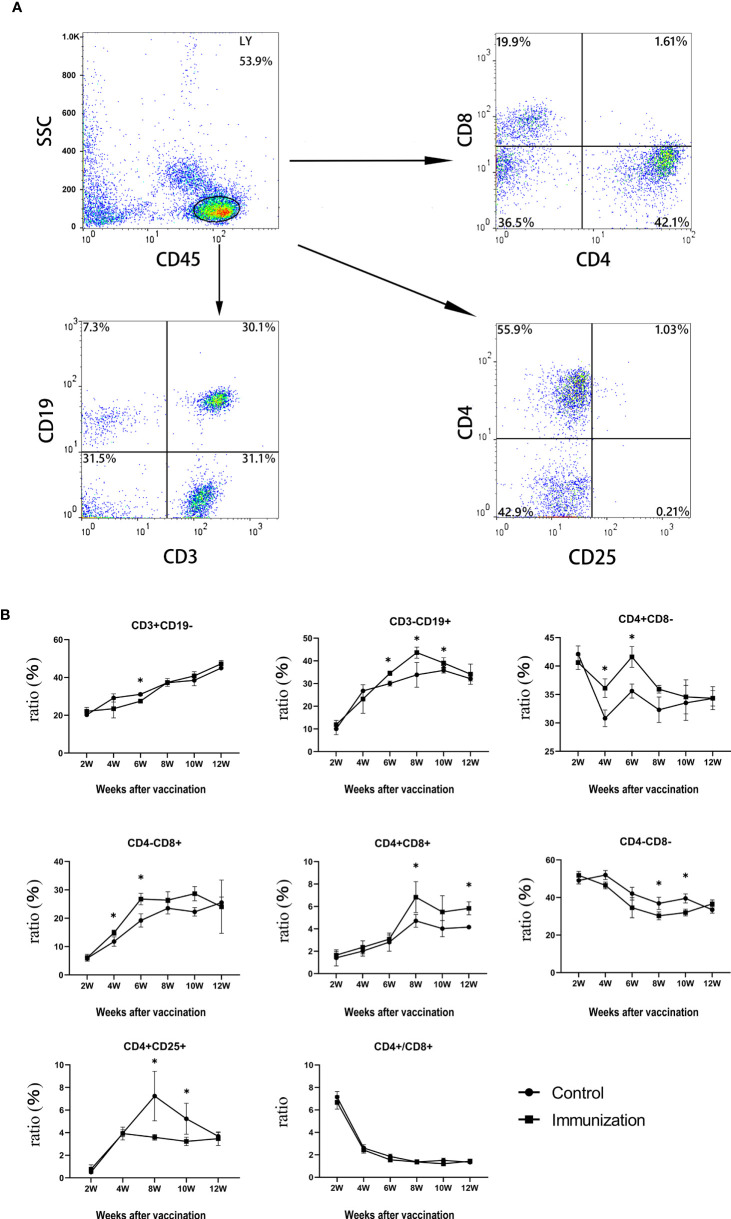
**(A)** The proportions of lymphocytes in blood of rats were separated and were analyzed by flow cytometry compared to control groups.Up left is CD45,up right is CD4 and CD8(CD45 for gate), down left is CD3 and CD19(CD45 for gate),down right is CD4 and CD25(CD45 for gate), **(B)** Mean (± SEM) of the serum CD3+CD19-、CD3-CD19+、CD4+CD8-、CD4-CD8+、CD4+CD8+、CD4-CD8-、CD4+CD25+ and the ratio of CD4+ and CD8+ (all from the gate of CD45+ cells)concentrations in the gonadotropin-releasing hormone (GnRH) immunization group (n = 35) and control group (n = 35). Flow cytometry was used to detect the number of immune cells. The ordinate is the ratio of immue cytokine. The abscissa is the age of weeks after first immunization. * Indicates a significant difference (*P*<0.05).

The percentage of some peripheral blood immune cells of rats in the immunized group and the control group were analysized by flow cytometry. **Figure 4A** shows the gating strategy. Within 6 weeks after the first immunization, the percentage of CD3+CD19- lymphocytes in the immunized group was significantly lower than that in the control group (*P < 0.05*). Thereafter, over time, the percentage of CD3+CD19- lymphocytes became similar between the immunized group and the control group (*P* > 0.05). The total percentage of T lymphocytes gradually recovered, and then slightly increased (**Figure 4B**, upper left panel). The percentage of CD4+ lymphocytes in the immunized group was significantly higher than that in the control group (*P <* 0.05, **Figure 4B**, upper right panel) from 4 to 6 weeks after the first immunization, and then became similar to that in the control group over time. The percentage of CD8+ lymphocytes in the immunized group was significantly higher than that in the control group from 4 weeks after the first immunization (*P <* 0.05, **Figure 4B**, middle left panel), and the change was similar to that of CD4+ lymphocytes. The percentage of CD4+CD25+ lymphocytes in the immunized group was significantly lower than that in the control group from 6 weeks to 10 weeks after the first immunization (*P* < 0.05, **Figure 4B**, lower left panel). The percentage of CD3-CD19+ lymphocytes in the immunized group was significantly higher than that in the control group from two weeks after the third immunization to 6 weeks after the third immunization (*P* < 0.05, **Figure 4B**, upper middle panel). The ratio of CD4+T cells to CD8+T cells decreased sharply during 2 weeks to 4 weeks after the first vaccination in both groups, and then gradually stabilized at about 2. But there was no significant difference between the two groups throughout the whole experimental period (*P* > 0.05, **Figure 4B**, lower middle panel).

### 3.5 Thymus and mRNA expression changes

**Figure 5:**
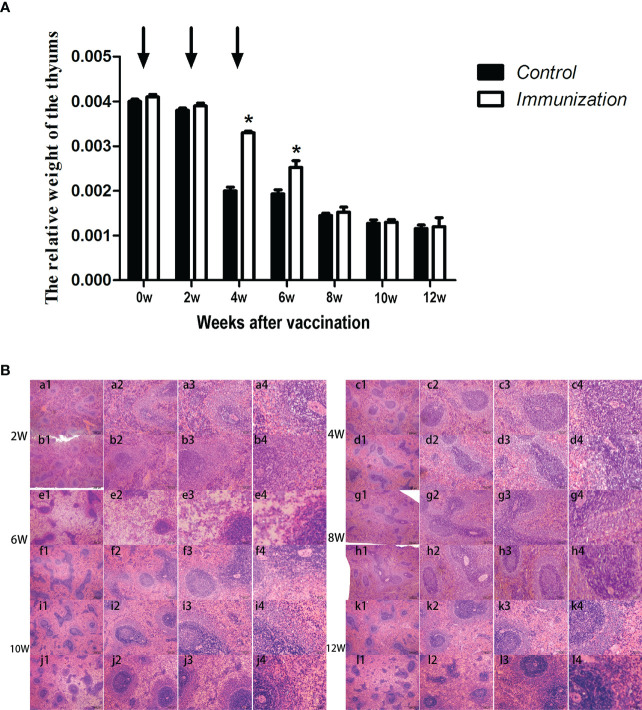
**(A)** Mean (± SEM) of the relative weight of the thymus in the gonadotropin-releasing hormone (GnRH) immunization group (n = 35) and control group (n = 35). Arrows indicate the primary vaccination and subsequent boosters. * Indicates a significant difference (*P* < 0.05). **(B)** Histopathological observation of the thymus of the control group (a, b, e, g, i, k) and gonadotropin-releasing hormone (GnRH) immunization group (b, d, f, h, j, l). All observations were performed on samples taken from 4 week old rats after first immunization. Numbers 1, 2, 3, and 4 represent a magnification of 40, 100, 200, and 400 times, respectively.

We found that, in both the immunized group and the control group, the relative weight of the thymus gradually decreased from 20 days old, and stabilized 8 weeks after the first vaccination (**Figure 5A**). However, the relative weight of the thymus in the immunized group was significantly higher than that of the control group at 4–8 weeks after the first immunization (*P < 0.05*). We observed the thymus after H.E. staining, which showed that the medulla had a light color, and the cortex was dark and dense. Over time, the thymus medulla gradually became larger in the control group, whereas, in the immunized group, the increase was smaller and the rate of change was slower (**Figure 5B**).

**Figure 6:**
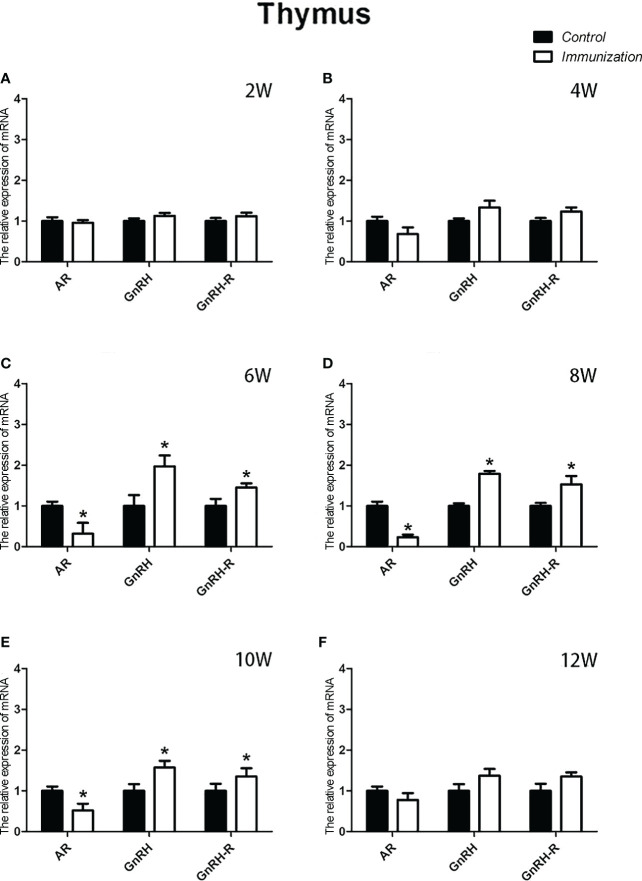
Mean (± SEM) of the effects of gonadotropin-releasing hormone (GnRH) immunization on the mRNA expression of reproduction-related genes in the thymus of the control (n = 35) and GnRH immunization (n=35) groups. **(A–F)** indicate the detection time at different ages. * Indicates a significant difference (*P* < 0.05).

**Figure 7:**
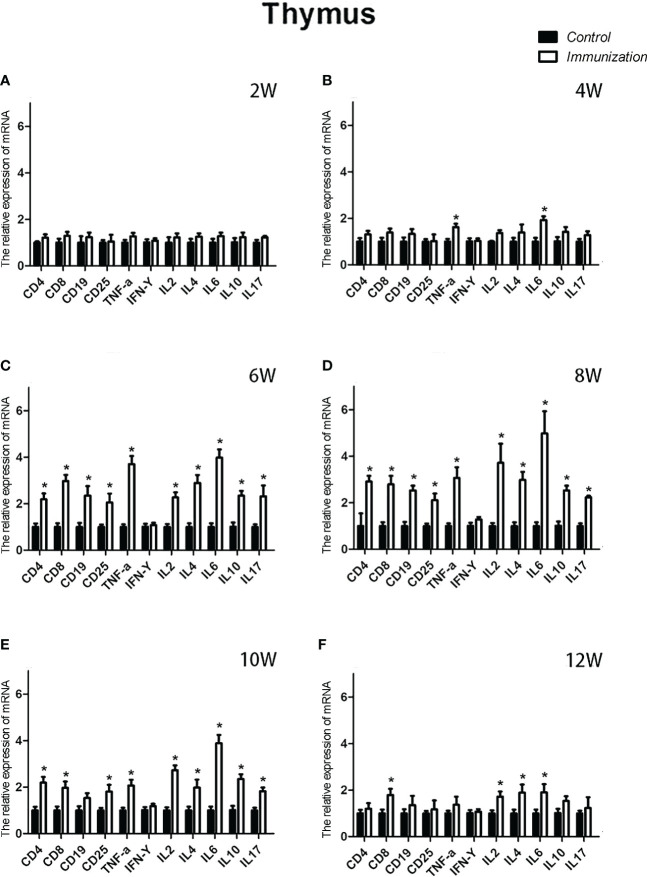
Mean (± SEM) of the effects of gonadotropin-releasing hormone (GnRH) immunization on the mRNA expression levels of immunity-related genes in the thymus of the control (n = 35) and GnRH immunization (n = 35) groups. **(A-F)** represent the detection time at different ages. * Indicates a significant difference (*P* < 0.05).

For the reproductive-related genes, the mRNA expression of *AR* in the immunized group was significantly lower than that in the control group at 6, 8, and 10 weeks after the first vaccination (*P* < 0.05, **Figure 6**), and the mRNA levels of *GnRH* and *GnRH-R* in the immunized group were significantly higher than those in the control group (*P < 0.05*, **Figures 6C-E**). For immune-related genes, there was no difference in the mRNA expression of *IFN-γ* between different periods (**Figures 7A-F**). During 6-10 weeks after the first vaccination, the mRNA expression of other immune genes was significantly higher in the immunized group than in the control group (*P < 0.05*, **Figures 7C-E**). In addition, the mRNA expression levels of *IL6* and *TNF-α* in the immunized group were significantly higher than those in the control group at 4 weeks after the first vaccination (**Figure 7B**). The mRNA expression levels of *CD8*, *IL2*, *IL4* and *IL6* in the immunized group remained significantly higher than that of the control group at 12 weeks after the first vaccination (**Figure 7F**).

### 3.6 Spleen changes and mRNA expression

**Figure 8:**
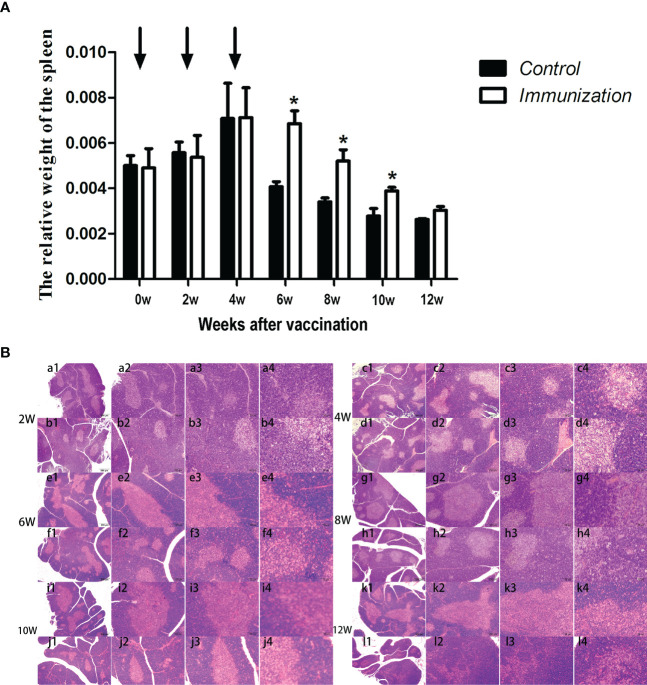
**(A)** Mean (± SEM) of the relative weight of the spleen in the gonadotropin-releasing hormone (GnRH) immunization group (n = 35) and the control group (n = 35). Arrows indicate primary vaccination and subsequent boosters. * Indicates a significant difference (*P* < 0.05). **(B)** Histopathological observation of the spleen of the control group (a, c, e, g, i, k) and the gonadotropin-releasing hormone (GnRH) immunization group (b, d, f, h, j, l). All observations were performed on samples taken from 4-week-old rats after first immunization. Numbers 1, 2, 3, and 4 represent a magnification of 40, 100, 200, and 400 times, respectively.

**Figure 9:**
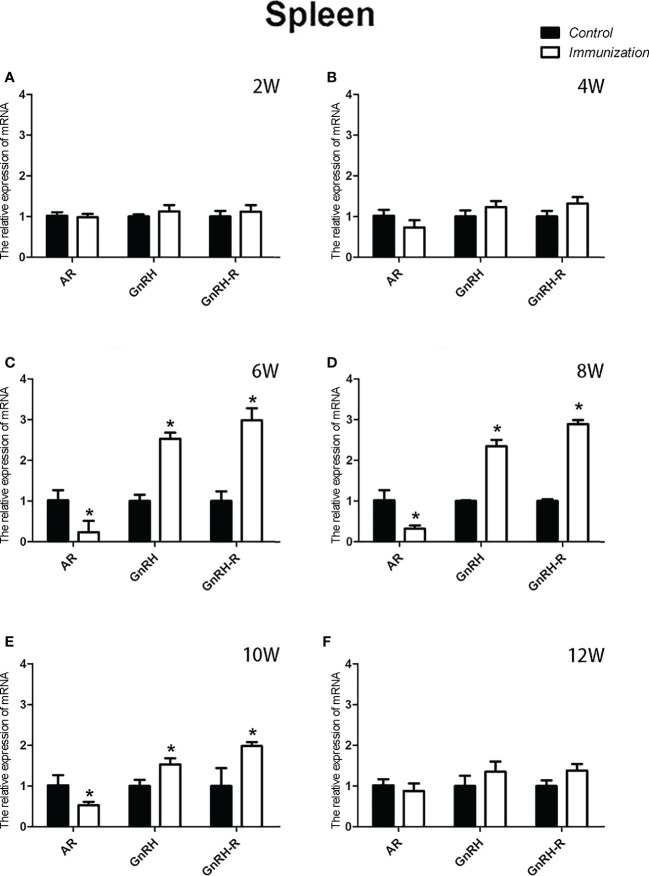
Mean ( ± SEM) effects of gonadotropin-releasing hormone (GnRH) immunization on the mRNA expression of reproduction-related genes in the spleen of the control (n = 35) and GnRH immunization groups (n = 35). **(A–F)** represent the detection time at different ages. * Indicates a significant difference (*P* < 0.05).

**Figure 10:**
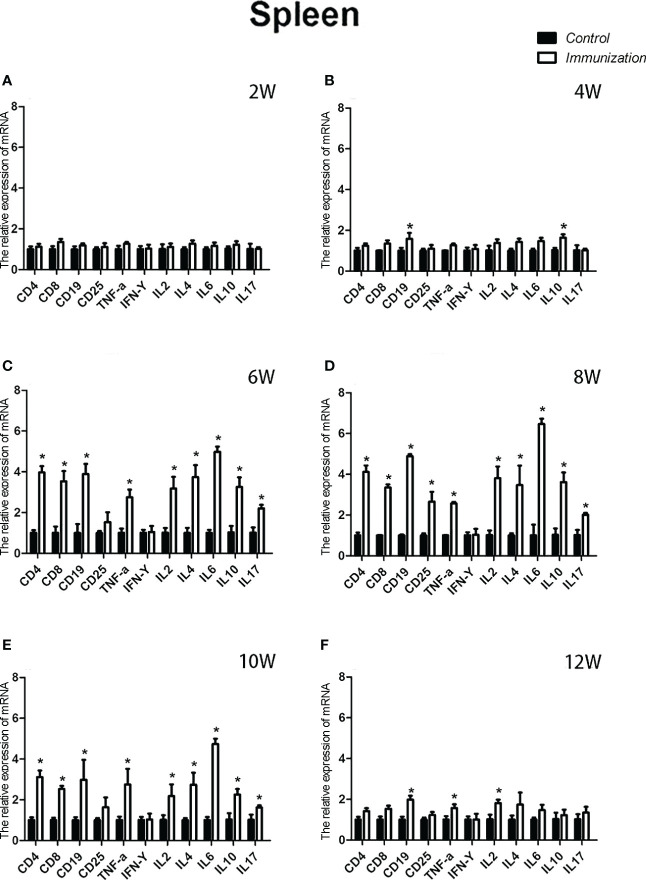
Mean (± SEM) of the effects of gonadotropin-releasing hormone (GnRH) immunization on mRNA expression of immunity-related genes in the spleen of the control (n = 35) and GnRH immunization groups (n = 35). **(A-F)** Indicate the detection time at different ages. * Indicates a significant difference (*P* < 0.05).

The relative weight of the spleen increased from 20 days old and reached a maximum at 4 weeks after the first immunization and then decreased in both the immunization and the control groups. However, the spleen weight of the immunized group was significantly higher than that of the control group from 6 –10 weeks after the first vaccination (*P* < 0.05, **Figure 8A**). We did not observe an obvious difference in the morphology of the spleens between the two groups after H.E. staining (**Figure 8B**). The expression of reproduction-related genes in the spleen showed the same trend as those in the thymus (**Figures 9A-F**). The mRNA expression of IFN-g was similar between the two groups (**Figures 10A-F**). The mRNA expression levels of other immune-related genes were significantly higher in the immunized group than in the control group from 6 –10 weeks after the first vaccination (*P < 0.05*, **Figures 10C-E**). The mRNA expression levels of *IL10* and *CD19* in the immunized group were significantly higher compared with those in the control group at 4 weeks after the first vaccination (*P < 0.05*, **Figure 10B**). The mRNA expression levels of *CD19*, *IL2*, and *TNF-α* in the immunized group were still significantly higher in the immunized group compared with that of the control group at 12 weeks after the first vaccination (*P < 0.05*, **Figure 10F**).

## 4 Discussion

In this study, we investigated the effects of immunocastration on the development and function of male rats’ reproductive system and immune system. In agreement with previous studies (36, 37, 42), GnRH active immunization stimulated the production of antibodies 2 weeks after the first immunization, and resultantly reduced the concentration of serum testosterone remarkably, inhibited the development of testes and spermatogenesis over time.

As a primary lymphoid organ for generating T cells, thymus begins to gradually atrophy in early stage of the life (43). Our results showed the similar process, but the progression of atrophy was remarkably delayed. Studies have confirmed that GnRH bears a stimulatory effect on the development and function of thymus. Blockade of GnRH receptor in neonatal rats inhibits the morpho-functional development of the thymus (26). GnRH agonist treatment stimulates the regeneration of the thymuses in old male rats (44), prevents lipopolysaccharide (LPS) induced thymic atrophy (34), and enhances T cells recovery following bone marrow transplantation (45). Since circulating GnRH in immunized rats was neutralized by the antibodies, so we speculated there must be some other factors or pathways account for the delayed thymic atrophy in immunized rats. It was reported that androgen exerted a negative effect on thymus. Ablation of androgen results in regeneration of thymus, restoration of peripheral T lymphocytes phenotype and function in elderly males and aged mice (46). Treatment with androgens causes atrophy (47). This is driven by signaling through the classical AR (48). A recent research demonstrates that GnRH exerts immunomodulatory effects mainly *via* autocrine or paracrine (49). So, we detected the mRNA expression of *GnRH, GnRH-R* and *AR* in thymus. Results showed that the mRNA expression of *GnRH* and *GnRH-R* was upregulated whereas that of *AR* was downregulated in thymuses in immunized rats during 4-10 weeks after the first immunization. These results combine with the reduced serum testosterone in immunized rats partially explained why GnRH immunization slowed the thymic atrophy. More experiment should be conducted to investigate, why the relative weight of thymus in the immunized rats was the same as that of the controls during 8-10 weeks after the first immunization, as the expression of reproduction-related genes (*GnRH, GnRH-R*, and *AR*) and the concentration of serum testosterone were the same as before.

A previous study in rams suggested that immunocastration against GnRH increased GnRH production in spleen and improved the immune markers of spleen and serum immune cytokines in rams (49). In the present study, we got similar results in male rats. Besides, in our experiment, the relative weight of spleen in immunization group were significantly higher than that of the control group during 6-10 weeks after the first vaccination, while there’s no difference between the immunized rams and the control rams. This difference may be caused by specie specificity.

Few studies have been carried out to investigate the effect of GnRH on the immune cells, and the role of GnRH in regulating immune cells remains elusive (50). Studies in rodents (51) and baboon (52) demonstrate that GnRH exerts stimulatory influences on B and T lymphocyte proliferation. However, studies in male mice confirm blood lymphocyte subsets showed a decreasing trend after GnRH agonist administration in pre-pubertal males, while no differences were observed in post-pubertal males (31). Immunization against GnRH didn’t alter the percentages of CD3+, CD3+CD4+ and CD3+CD8+ lymphocytes in male pigs (53), wheares, increased the number of CD3+ and CD4+ T lymphocytes initially in the peripheral blood, and eventually restored the T lymphocytes to normal levels in rats (37). GnRH antagonist treatment decreased the percentage of CD4+CD25+ cells, reduced mitogen-induced CD8 T cells IFN-γ expression, but didn’t affected the ratio of CD4 to CD8 T cells in adult males (32). In this study, we found the GnRH immunization decreased the percentage of CD3+ and CD4+CD25+ T cells, and increased the percentages of CD4+ and CD8+ cells without affecting the ratio of CD4+ to CD8+ T cells. CD25 is expressed on the surface of both immune cells and non immune cells (54), only CD4+CD25hi are T-regulatory cells (T-regs) (55). CD4+CD25hi Treg cells are potent suppressors of the proliferation of CD4+CD25− and CD8+ T-cells (56–58) and the inhibition effects are mediated mainly *via* cell-cell contact and possibly *via* secretion of soluble immune suppressive cytokines to suppress the response of effector cells to self-antigens or exogenous antigens (protective immunity) (59, 60). In this experiment, the percentage of CD4+CD25+decreasing accompanied the percentage of CD4+ and CD8+ cells and serum immune factors (IL-2, IL-4, IL-6, IL-10, and TNF-α) increasing in GnRH immunized rats. This implies that GnRH immunization may, at least partly, enhance the body immunity by inhibiting the development of CD4+CD25+ T-regs. It may be that the testosterone level decreases after GnRH immunization, causing changes in the thymus (61) and splenic, and then cause the change of CD4+CD25+cell proportion (62, 63).This result is the same as the effect of medical castration on CD4+CD25+T cells (32). Here, we found except CD3+ cells were downregulated during initial stage, and CD4-CD8- cells were downregulated throughout the experiment, the profiles of peripheral blood lymphocytes including CD3-CD19+, CD3+CD19-, CD4-CD8+, CD4+CD8+, and CD4-CD8-, as well as the mRNA expression of their markers were increased in thymuses and spleens at different time point after immunization. The ratio of CD4+and CD8+ cells decreases with age is a normal phenomenon (64). This could be ascribed to the age-related decline in thymopoietic efficacy (65). Here, the percentage of CD4+ and CD8+ T cells in lymphocytes increased and their trends are similar after GnRH immunization, which led to no significant increase in the ratio of CD4 and CD8 compared with the control group.The reason for these differences is that, on the one hand, GnRH immunity achieves immune regulation by reducing serum sex hormone levels, while estrogen and androgen have opposite immune effects (66, 67); On the other hand, it may be related to the physical state of animals. Therefore, it is speculated that in this study, because the male rats are in a healthy state, the decrease in the total number of T lymphocytes is a direct response of immune cells to changes in the endocrine axis in the early stage after GnRH immunization (36). However, because immunity has a long-term effect, the total number of T lymphocytes in the body eventually returns to normal as the body’s immune system gradually adapts to the changes (37). Whatever, the exact role and the mechanism of GnRH on remains to be investigated.

The concentrations of IL-2, IL-4, IL-6, IL-10 and IL-17 in the serum of rats were significantly increased after immunization with GnRH. Interferon-γ, interleukin-2, and tumor necrosis factor-α are secreted by Th1 cells and play important roles in cellular immunity. IL-4, IL-6 and IL-10 are secreted by Th2 cells (68, 69), which can stimulate the proliferation of B lymphocytes and produce specific antibodies to assist humoral immunity (70, 71). Th17 cells, a newly identified subset of T lymphocytes, can secrete IL-17, which is involved in autoimmune diseases and defense responses (72). All play an important role. From the relevant data of this experiment, the change trend of immune cytokines and reproductive hormones is opposite. From the second immunization, the cytokines and reproductive hormones of the immunized group were significantly different from those of the control group for a period of time. So, immunization with GnRH can enhance the humoral and cellular immune responses in rats within a certain period of time after immunization. In addition, tumor necrosis IFN gamma was unaffected. In most studies, the effects of active immunization with GnRH on the immune system were not permanent (37). The antibody concentration did not recover during the entire experiment. These results are consistent with the present experiment.

## 5 Conclusion

Taken together, our data suggest that GnRH immunization can increase the activity of the GnRH/GnRH-R receptor signaling pathway in the thymus and spleen by suppressing feedback, thereby reducing the content of androgens, especially testosterone. This ultimately resulted in increased immune markers in the thymus, spleen, and blood of rats. however, this effect disappears, as time goes on. That is to say, immune castration can temporarily improve the immunity of immunized animals to a certain extent on the basis of inhibiting the reproductive ability of male animals.

## Data availability statement

The original contributions presented in the study are included in the article/**Supplementary Material**. Further inquiries can be directed to the corresponding authors.

## Ethics statement

The animal study was reviewed and approved by Animal Care and Use Committee of Anhui Agricultural University.

## Author contributions

YSL, FF, YHL, YHZ and YL : Resources, supervision, project administration, and funding acquisition. WT: Data curation, original draft writing and preparation. FP: Original draft writing and preparation, manuscript review and editing. HD: Investigation, and data curation. HX, BZ, and WF: Data analysis and validation. All authors contributed to the article and approved the submitted version.

## Supplementary material

The Supplementary Material for this article can be foundonline at: https://www.frontiersin.org/articles/10.3389/fimmu.2022.1023104/full#supplementary-material
