# Supplementary material for: Effect of GnRH immunocastration on immune function in male rats
Source: Front Immunol. 2023 Jan 13;13:1023104. doi: 10.3389/fimmu.2022.1023104 (PMC9880316; doi:10.3389/fimmu.2022.1023104)
Supplement: Supplementary file 1 [file Table_1.docx]

Supplementary table

Table 1

| **Gene** | **GenBank accession No.** | **Primer sequence (5′-3′)** | **Amplification length (bp)** |
| --- | --- | --- | --- |
| ***GnRH*** | [**NM_012767.2**](https://www.ncbi.nlm.nih.gov/nuccore/NM_012767.2) | **F:TGGTATCCCTTTGGCTTTCACA**  **R:TGATCCTCCTCCTTGCCCAT** | **193** |
| ***GnRH-R*** | [**NM_031038.3**](https://www.ncbi.nlm.nih.gov/nuccore/NM_031038.3) | **F:CTAACAATGCGTCTCTTGA**  **R:TCCAGATAAGGTTAGAGTCG** | **140** |
| ***AR*** | [**NM_012502.1**](https://www.ncbi.nlm.nih.gov/nuccore/NM_012502.1) | **F:GGCAGTCATTCAGTATTCC**  **R:AGTAGAGCATCCTAGAGTTG** | **172** |
| ***CD4*** | [**NM_012705.1**](https://www.ncbi.nlm.nih.gov/nuccore/NM_012705.1) | **F:GAGTTGAGATGGAAGGCAGAG**  **R:GTGAGTGGGAGCGTTTCG** | **135** |
| ***CD8*** | [**XM_008762976.2**](https://www.ncbi.nlm.nih.gov/nuccore/XM_008762976.2) | **F:GTGGAGGGAATGGGATTGG**  **R:AGCAGATGAGAGTGATGACC** | **135** |
| ***CD19*** | [**NM_001013237.3**](https://www.ncbi.nlm.nih.gov/nuccore/NM_001013237.3) | **F:AGGAGGAAGAGGAAGCGAATG**  **R:GCCAGAGGTAGATGTAGGAAGG** | **157** |
| ***CD25*** | [**NM_013163.1**](https://www.ncbi.nlm.nih.gov/nuccore/NM_013163.1) | **F:GCTGGTCTATATGGCTTGTC**  **R:TGTTGATTTCTGCGTGTCC** | **101** |
| ***TNF-α*** | [**NM_012675.3**](https://www.ncbi.nlm.nih.gov/nuccore/NM_012675.3) | **F:TCAGCCTCTTCTCATTCC**  **R:ACTTCTCCTCCTTGTTGG** | **153** |
| ***IFN-γ*** | [**NM_138880.2**](https://www.ncbi.nlm.nih.gov/nuccore/NM_138880.2) | **F:TACGACATTTCCGACTG**  **R:CTTGTGCTTCACCCTAA** | **190** |
| ***IL2*** | [**NM_053836.1**](https://www.ncbi.nlm.nih.gov/nuccore/NM_053836.1) | **F:GCGCACCCACTTCAAGCCCT**  **R:CCACCACAGTTGCTGGCTCA** | **148** |
| ***IL4*** | [**NM_201270.1**](https://www.ncbi.nlm.nih.gov/nuccore/NM_201270.1) | **F:CCCCCAGCTAGTTGTCATCC**  **R:GTTGCTGTGAGGACGTTTGG** | **108** |
| ***IL6*** | [**NM_012589.2**](https://www.ncbi.nlm.nih.gov/nuccore/NM_012589.2) | **F:TCCAGCCAGTTGCCTTCTTG**  **R:GGTCTGTTGTGGGTGGTATCC** | **127** |
| ***IL10*** | [**NM_012854.2**](https://www.ncbi.nlm.nih.gov/nuccore/NM_012854.2) | **F:AGTGGAGCAGGTGAAGAATG**  **R:CCAGCCTTAGGATCGAAGTT** | **188** |
| ***IL17*** | [**NM_001106897.1**](https://www.ncbi.nlm.nih.gov/nuccore/NM_001106897.1) | **F:CTCAACCGTTCCACTTCACC**  **R:CACTTCTCAGGCTCCCTCTTC** | **127** |
| ***β-actin*** | [**NM_031144.3**](https://www.ncbi.nlm.nih.gov/nuccore/NM_031144.3) | **F:GCGCAAGTACTCTGTGTGGA**  **R:AAGGGTGTAAAACGCAGCTC** | **160** |

Table1: GnRH, gonadotropin-releasing hormone; GnRH-R, gonadotropin-releasing hormone receptor; AR, androgen receptor; CD4, CD4 molecule; CD8, CD8 molecule; CD19, CD19 molecule; CD25, CD25 molecule; TNF-α, tumor necrosis factor alpha; IFN-γ, interferon-γ; IL-2, interleukin 2; IL-4, interleukin 4; IL-6, interleukin 6; IL-10, interleukin 10; IL-17, interleukin 17; β-actin, beta actin.
